# Summer "Sickness and Diarrhœa."

**Published:** 1912-06-29

**Authors:** J. H. Heaney


### SUMMER "SICKNESS AND DIARRHŒA."

### The Treatment of the Disease in Children.

By J. H. HEANEY, M.B.

For this purpose it will be well to divide these cases into two classes: (1) Those in which the diarrhœa is complicated with excessive and obstinate vomiting, and (2) those in which vomiting is absent or altogether of secondary importance.

In the first class the child, especially if very young, is threatened with sudden collapse.

The room should be well ventilated and hot applications packed round the abdomen and lower extremities. If hot fomentations are used it is advisable and useful to smear the parts beforehand with olive oil or some greasy substance. In this way the skin is less likely to be injured.

Anything by the mouth should only be given in teaspoonfuls and at regulated intervals. I have found most useful of all five to ten drops of Carnrick's liquid peptonoids in a teaspoonful of water every five or ten minutes.

If the restlessness is moderate or great a small dose of Dover's powder ought to be given as soon as there seems a chance of its being retained—and this at any age. This may sometimes, with advantage, be replaced by a drop or more according to age of liq. morph. hydroch., plus a little iced water placed in the mouth.

Citrate of soda freshly prepared ought to be given every one or two hours. The size of the dose is not very important. As the vomiting lessens one can go on with albumen water for twenty-four hours, gradually increasing the quantity.

By this time the diarrhœa ought to be improving, and a bismuth mixture may be all that is needed to complete the cure. The salicylate of bismuth shaken up with water is, I think, more useful than the soluble preparations now on the market. But the carbonate, as recommended long ago by Hutchinson, does very well.

If the motions are still very watery, a small dose of pulv. kino co., which may be repeated, is most useful. Often the excessive loss of fluid has to be combated, and active measures ought always to be taken if the skin shows any loss of elasticity. Subcutaneous injections of saline are better than rectal, except the latter can be administered continuously. But there are often difficulties in the way of this, and the same may be said of washing out the stomach, which is so well spoken of in cases of vomiting. However, in very young and collapsed children I would prefer the method I have indicated.

Why peptonoids should be so useful in practice and hardly to be recommended in theory is difficult to say, but their administration has undoubtedly an excellent effect on the vomiting. Opium, I think, is given too sparingly in many of these cases. There are no definite lines to be laid down as regards dose. Each case must be considered individually. Isolated doses only should be employed.

